# Amoebicidal Activity of Essential Oil of *Dysphania ambrosioides* (L.) Mosyakin & Clemants in an Amoebic Liver Abscess Hamster Model

**DOI:** 10.1155/2014/930208

**Published:** 2014-03-16

**Authors:** Manuel Enrique Ávila-Blanco, Martín Gerardo Rodríguez, José Luis Moreno Duque, Martin Muñoz-Ortega, Javier Ventura-Juárez

**Affiliations:** ^1^Autonomous University of Aguascalientes, 20131 Aguascalientes, AGS, Mexico; ^2^Physiology and Pharmacology Department, Basic Science Center, Autonomous University of Aguascalientes, 20131 Aguascalientes, AGS, Mexico; ^3^Chemistry Department, Basic Science Center, Autonomous University of Aguascalientes, 20131 Aguascalientes, AGS, Mexico; ^4^Morphology Department, Basic Science Center, Autonomous University of Aguascalientes, 20131 Aguascalientes, AGS, Mexico

## Abstract

Amebiasis is a parasitic disease that extends worldwide and is a public health problem in developing countries. Metronidazole is the drug recommended in the treatment of amebiasis, but its contralateral effects and lack of continuity of treatment induce low efficiency, coupled with the appearance of resistant amoebic strains. Therefore, the search of new compounds with amoebicidal activity is urgent and important. In this study, we evaluated the in vitro and in vivo antiamoebic activity of the essential oil *Dysphania ambrosioides* (L.) Mosyakin & Clemants. It exhibited an IC_50_ = 0.7 mg/mL against trophozoites. The oral administration of essential oil (8 mg/kg and 80 mg/kg) to hamster infected with *Entamoeba histolytica* reverted the infection. Ascaridole was identified as the main component of essential oil of *D. ambrosioides*. The identification of amoebicidal activity of Ascaridole gives support to the traditional use. Further studies with Ascaridole will be carried out to understand the mechanism involved.

## 1. Introduction

Amebiasis is the infection of human gastrointestinal tract caused by* Entamoeba histolytica,* a protozoan parasite capable of invading the intestinal mucosa and that may spread to other organs, mainly the liver which usually leads to amoebic liver abscess. Spread occurs via the fecal-oral route, generally, by poor hygiene during food preparation and eating or by the use of human waste as fertilized crops. Crowding and poor hygiene contribute to its prevalence in Africa, Asia, and Latin America. Fifty millions of people suffer from amebic colitis y/o liver abscess caused by* Entamoeba histolytica* resulting in 50,000 to 100,000 deaths yearly [1]. For example, it was observed that 39% of children from an urban slum in Dhaka, Bangladesh, had a new* E. histolytica* infection during 1-year study. This infection remains a significant cause of morbility worldwide [2]. Among children, under 5 years who were admitted with acute diarrhea in a hospital,* Entamoeba histolytica* was confirmed in 7.8% of the cases [3]. The estimated number of infected cases may be much higher due to asymptomatic diseases and lack of a sensitive and specific diagnostic test [4].

Metronidazole is commonly used and recommended as the treatment of amebiasis [5]. However, it is less effective in the tissue than in the gut lumen [6]. In addition, it can eradicate only up to 50% of laminae infections, and this drug has been reported to have unpleasant side effects such as metallic taste, headache, dry mouth, and to a lesser extent nausea, urticaria, pruritus, and dark-colored urine [7–9]. Thus, the search for alternative antiamebic compounds with high activity, cheap, and more effective is still a necessary goal.

*Dysphania ambrosioides* (L.) Mosyakin & Clemants before known as* Chenopodium ambrosioides* traditionally named “Epazote” is a herb native to South America. It is also cultivated in subtropical and subtemperate regions, mostly for consumption as leafy vegetable and herb. Because of the pungent flavor, it is traditionally used to flavor beans and other South American dishes [10, 11]. Herb is a plant widely known in popular medicine as anthelmintic, vermifuge, and emmenagogue [10, 12]. It is used in the treatment of digestive, respiratory, urogenital, vascular, and nervous disorders. Kumar et al. [13] described the antifungal activities of* Chenopodium* oil against eight fungi including: Aspergillus niger, Aspergillus fumigatus, Botryodiplodia theobromae, Fusarium oxysporum, Sclerotium rolfsii, Macrophomina phaseolina, Cladosporium cladosporioides and Helmintosporium oryzae [10, 13]. Ascaridole is the main active compound found in the essential oil [14–16]. It was found to be a potent inhibitor in vitro development of* Plasmodium falciparum*,* Trypanosoma cruzi,* and* Leishmania amazonensis* [17].* Chenopodium ambrosioides *continues to be used widely to treat intestinal disorders and other illness in humans, with apparent success [18]. Although that chemical composition of Epazote is known, amoebicidal properties have not been linked to specific essential oil. In this study, we report the amoebicidal activity of Mexican Epazote against* Entamoeba histolytica*, by assessing its effect in vitro and in vivo on the model of amebic liver Abscess induced in the hamster [19].

## 2. Material and Methods

### 2.1. Plant Material

Epazote leaves were bought from a local market (“Central Comercial Agropecuario,” Aguascalientes, Lat 21.91117, Long 102.28984). A voucher specimen (number 7607, Ph.D. Maria Elena Siqueiros Delgado) was deposited at the botanical garden at Autonomous University of Aguascalientes. The dried plant material was grounded and mixed 1 : 5 with water and subjected to stream distillation for 3 hours at the atmospheric pressure. The distillate was extracted twice with dichloromethane (1 : 1 v/v). The organic phase was collected and dried with anhydrous sodium sulfate; dichloromethane was evaporated in a rotator evaporator at 30°C under reduced pressure and weighted. The hydrodistillation of the plant yielded clear oil with characteristic odor.

*Animals*. Golden male hamsters (*Mesocricetus auratus*) from six to eight weeks old (weight 100–160) were maintained in cycles of light/dark (12 : 12); food was supplied from Ralston Rations-Kansas (Purina) and water* ad libitum* in the animal facility of the Autonomous University of Aguascalientes. The animal use protocol that was approved by the Ethics Committee of the Autonomous University of Aguascalientes.

### 2.2. Essential Oil Analysis

The compounds in the essential oil of Epazote were tentatively identified by gas chromatography combined with mass spectrometry (GC-MS). Mass spectral data were obtained on a gas chromatograph-mass spectrometer (Agilent GC model 6850, Agilent MS model 5975C). Fused capillary columns HP5-MS (30 m × 0.25 mm; film thickness of 0.25 *μ*m). The GC oven temperature was programmed from 60 to 200°C with increments 15°C/min. One microliter of the sample was injected by the split mode (10 : 1) with the split vent being closed for 30 s. Helium was the carrier gas at flow of 2 mL/min. The mass spectrometer was scanned from* m/z* 30 to 500 UM in electron impact mode (70 eV), and chromatogram time was 10 min. The identification of individual components was based on their spectral fragmentation according to the mass spectra library Wiley, retention indices, and comparison with published data. It only compounds with similarity indexes of 90% that were considered as positive identifications.

### 2.3. *Entamoeba histolytica* Culture

*Entamoeba histolytica* HM-1 : IMSS strain was used for all experiments; the trophozoites were cultured axenically in screw-cupped tubes at 35.5°C on Diamond's TYI-S-33 medium, supplemented with 10% (v/v) heat-inactivated bovine serum. Subcultures were performed routinely at 48 hr intervals by replacing the medium without detaching the monolayer. Cells were harvested by replacing the medium with fresh one, chilling on ice for 20 min, and inverting gently to detach the monolayer.

### 2.4. Measurement of Amoebic Activity In Vitro

The Epazote essential oil (EEO) was sterilized through a 0.22 *μ*m Millipore filter and then transferred to sterile Eppendorf tube. Different concentrations of essential oil (0.05, 0.1, 0.25, 0.5, 0.75, 1.0, 1.25, and 1.5 mg/mL) and metronidazole (10 *μ*g/mL as standard amoebicidal) were prepared in test tubes by serial dilutions in sterile water. Diamond's TYI-S-33 medium was added to the test tubes. Subsequently, 1.0 × 10^3^ trophozoites of* Entamoeba histolytica* were added to the test tubes and then incubated at 36°C for 48 h. After this time, the tubes were chilled for 20 min, and 1 mL of the medium was taken off from each tube for the viability count using trypan blue exclusion technique. Motility and dye exclusion were the criteria for viability. The percentage of growth inhibition was calculated using the following formula: % GI = (1 − Extract/Control) × 100. The experiments were performed in triplicate and repeated three times.

### 2.5. Measurement of Amoebic Activity In Vivo

Twenty-five male golden hamsters (*Mesocricetus auratus*) were divided into five groups of five animals each. First, group was considered as intact control, and other groups were inoculated with 30–40 × 10^4^ trophozoites in the central hepatic lobe to induce amoebic liver abscess (ALA) [19, 20]. Second group was called positive control, the third and fourth groups were treated with EEO to 8 mg/kg and 80 mg/kg, the fifth group was treated with metronidazole 0.1 mg/kg daily administered orally for 7 days after inoculation of amebas, and then hamsters were sacrificed. Livers were dissected and weighted, and representative tissue samples were fixed in 10% neutral formalin. Tissue liver slides were stained with haematoxylin-eosin and Masson trichrome methods [21]. The identification of trophozoites in amoebic liver abscesses was carried out by antibody versus Lectin 220 Kd (Kindly donated by Dr. Talamás-Rohana).

### 2.6. Statistical Analysis

The values were expressed as average ±S.D. The results were analyzed by Analysis of Variance (ANOVA) followed by the Tukey's test. The analyses were performed with the software GraphPad Prism 5.0.4. Differences were considered significant at *P* < 0.05. The IC50 estimation was evaluated using the logistic-curve model with GraphPad Prism 5.0.4 software.

## 3. Results and Discussion

**Figure 1:**
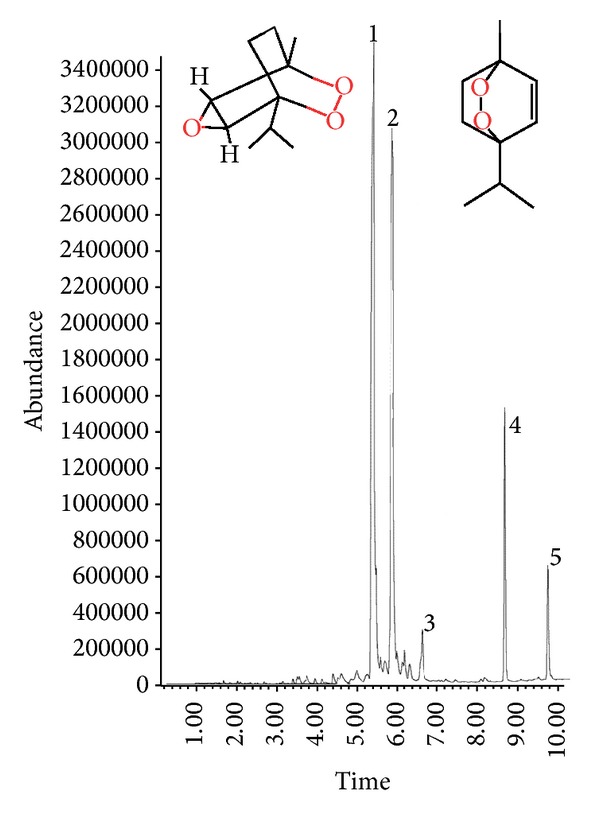
Reconstructed gas chromatogram of* Dysphania ambrosioides* (L.) Mosyakin & Clemants essential oil; peak numbers are the identified compounds: (1) Ascaridole endoperoxide; (2) cis-Ascaridole; (3) 7-oxabycilco (4.1.0) heptan 2-one, 3 methyl-6-(1-methyl-ethyl); (4) 2-propenoic acid, 2 methyl, dodecyl ester; (5) Methacrylic acid, tetradecyl ester.

Hydrodistillation of the Epazote plant yielded 1.0% essential oil on a fresh weight basis. Through GC-MS and chemical analysis, a total of 5 components were identified in essential oil, accounting for 93.2% of this total oil. The main constituents of the essential oil were Ascaridole epoxide (45.5%), cis-Ascaridole (34.2%), 7-oxabyciclo(4.1.0) Heptan 2-one, 3 methyl-6-(1-methy-ethyl) (2.5%), 2-propenoic acid, 2-methyl, dodecyl ester (7.2%), and Methacrylic acid, tetradecyl ester (3.54%) (Figure 1). The cis-Ascaridole and Ascaridole epoxide account at the 79% of essential oil. The results of the chemical composition of essential oil of* Dysphania* are in the category of rich oils in ascaridole [22] and it is agreed to the study published by Jardim et al. [10]. The chemical characterization of the essential oil of the 13 species medicinal in the genus* Chenopodium*, too, identified cis-Ascaridole as a major compound with range from 4.2% to 46.9% [15, 23]. Ascaridole epoxide has been described as a metabolite in the synthesis pathway of Ascaridole. Dembitsky et al. [15] mentioned that the sum of Ascaridole-related compounds is a more accurate quantitative, and the total content can be obtained by GC-MS analysis.

**Figure 2:**
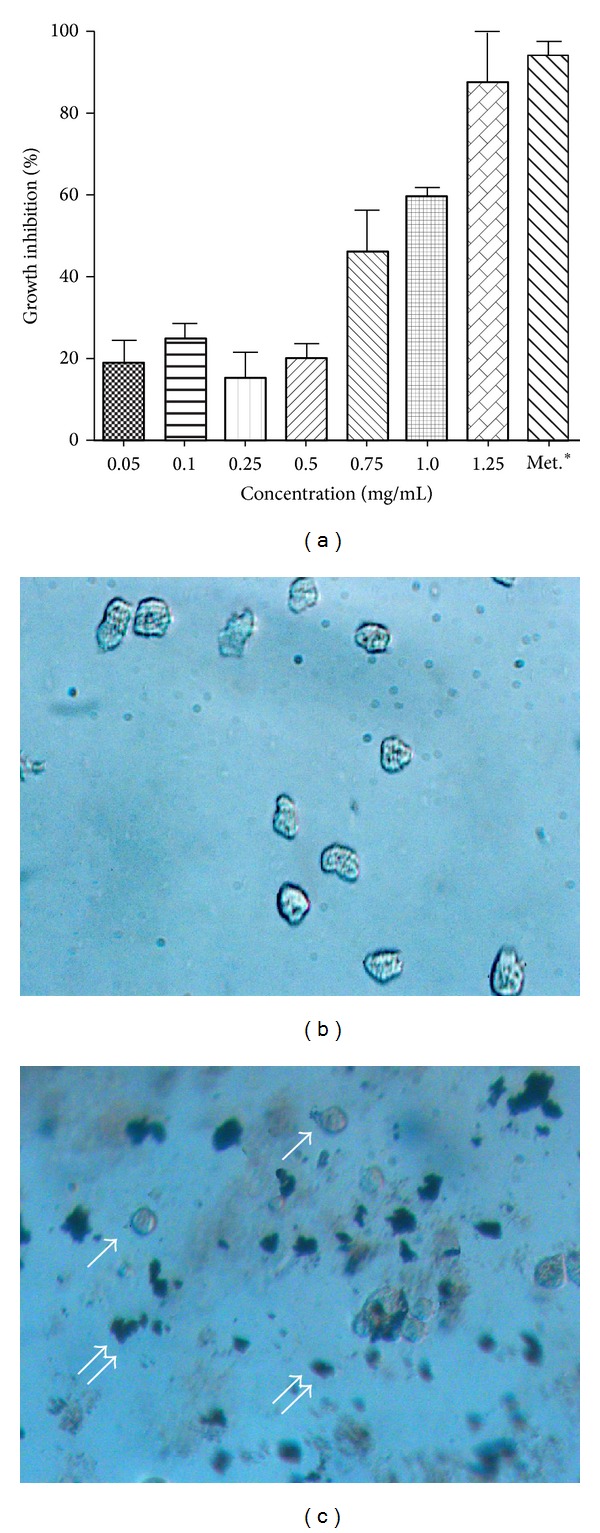
(a) Effects of essential oil of* Dysphania ambrosioides* (L.) Mosyakin & Clemants*. ambrosioides* on growth inhibition of* Entamoeba histolytica* in vitro. The last bar illustrates trophozoites after their exposure to metronidazole; (b) photography of control trophozoites, they were found attached to the support; and (c) trophozoites exposed to essential of* Dysphania ambrosioides* (L.) Mosyakin & Clemants were observed spheric form (single arrow) and amorphous mass (double arrow).

The EEO amebicidal ability was assayed using trypan blue exclusion; the viable trophozoites of* Entamoeba histolytica* remained clear in the control, whereas trophozoites exposed to metronidazole or EEO were light blue in color. Incubation of* Entamoeba histolytica* trophozoites in the range 0–1.25 mg/mL of essential oil caused growth inhibition, many trophozoites were observed suspended within the tube after incubation time, and they showed spherical form and amorphous mass without amoebic movement (Figures 2(b) and 2(c)). When the concentration range increased, the number of living trophozoites decreased (Figure 2(a)). The EEO showed an IC50 of 0.75 mg/mL; from this result, two doses of 10 and 100 higher than IC50 were choice for testing the antiprotozoal activity by an in vivo study. Essential oil from Epazote had amoebicidal activity dose-dependent. Although, some medicinal plants have been found with antiamoebic effect in vitro, there are a few data about the Epazote related to* Entamoeba histolytica*; thus, Calzada et al. [24] have reported that methanolic extract of* Chenopodium ambrosioides* showed a moderate activity toward* Entamoeba histolytica*. In the same way, other plants have been antiamoebic activity, including* Codiaeum variegatum* [25],* Thymus vulgaris* [26], and* Chiranthodendron pentadactylon* [24]. The essential oil of genus* Chenopodium* has displayed great activity against intracellular parasites, including* Trypanosoma cruzi*,* Plasmodium falciparum,* and* Leishmania amazonensis* [17, 27, 28].

**Figure 3:**
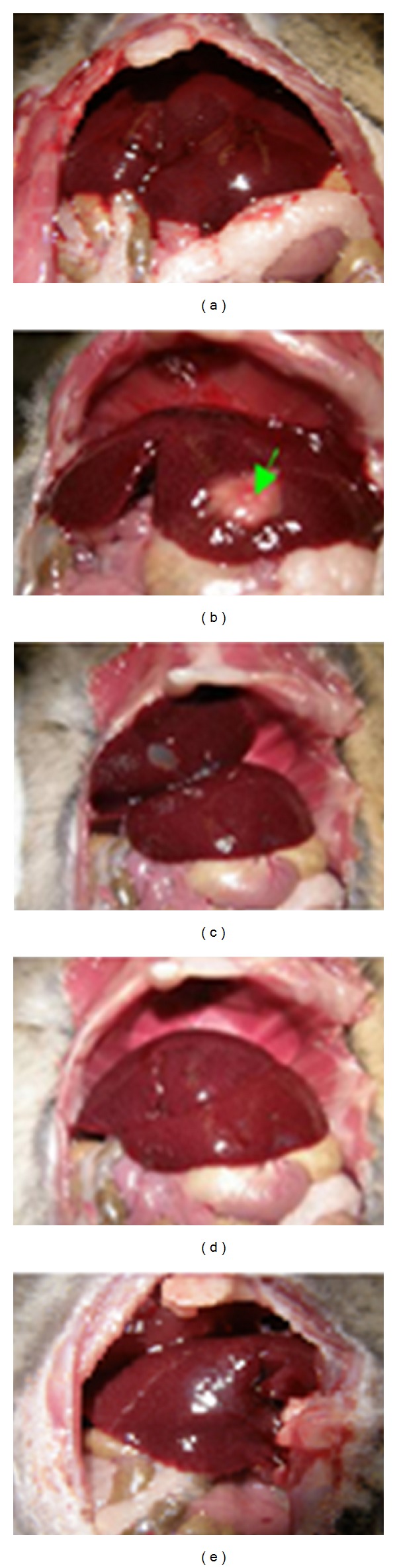
Photographies of hepatic abscesses induced by* Entamoeba histolytica* after seven-day after inoculation: (a) control; ((a)–(e)) inoculated with trophozoites; (c) and (d) treated with* Dysphania ambrosioides* (L.) Mosyakin & Clemants at 8 and 80 mg/kg, and (e) treated with metronidazole.

**Figure 4:**
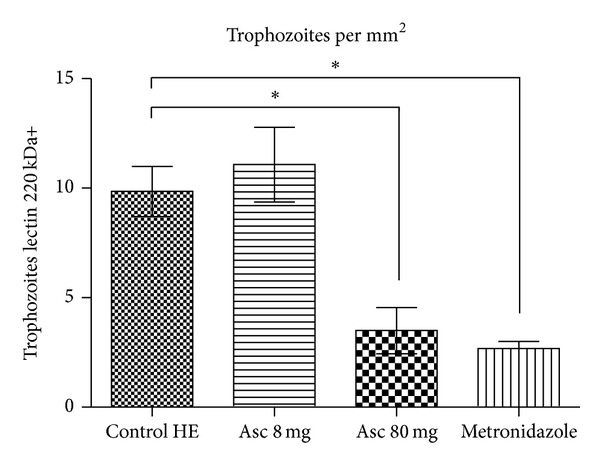
Account of trophozoites in amoebic liver abscess. We observe that the treatment with 80 mg of Ascaridole (asc) decreased significantly the number of trophozoites similar to metronidazole treatment; *P < 0.05.

**Figure 5:**
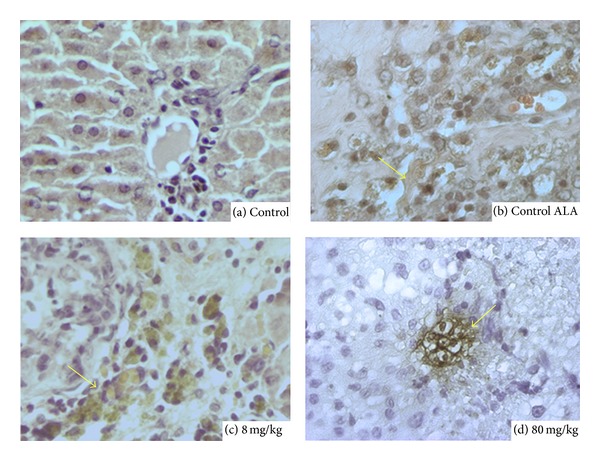
Amoebas marked with 220 kDa lectin during ALA 7 days after inoculation. (a) Normal histology of hamster liver (control). (b) Amoebic liver abscess after 7-day inoculation. Hamsters showed fragmented amoeba (arrow), fibrosis, and inflammatory infiltrate at the lesion. (c) Hamsters treated with 8 mg/kg of Ascaridole showed less fibrosis and small granulomas (arrow), whereas (d) hamsters treated with high Ascaridole dose had lesion by* Entamoeba histolytica* (arrow) reduced, without inflammatory infiltrate, reverted completely.

Most plant studies have been addressed to test the amoebicidal activity in vitro [24–26]; however, few studies, if any, have been done to examine the amoebicidal effects of Epazote in vivo amoebic liver abscess. Seven days after* Entamoeba histolytica* inoculation, there was a significance difference in liver weights in three experimental groups: ALA, EEO 8 mg + ALA, and EEO 80 mg + ALA (4.67 ± 0.42 g, 0.17 ± 0.5, and 0.04 ± 0.05 g, *n* = 5, resp., Figure 3). The animals inoculated with* Entamoeba histolytica* developed abscesses as well as the presence of multiple granulomas (Figure 4(b)). However, hamsters treated with essential oil of Epazote 8 mg daily after amebic inoculation had developed smaller abscesses, and two hamsters treated with 80 mg developed abscesses. In contrast, only one animal treated with metronidazole showed ALA. The quantity of amoebas in area of the abscess was estimated by immunohistochemistry with an antibody for ameba Lectin 220 kDa. Hamsters inoculated with* Entamoeba histolytica* had 9.8 ± 2 amebas/mm^2^. Hamsters treated with 8 mg of EEO was not different from control (11.1 ± 3.4 amebas/mm^2^); however, animals treated with EEO 80 mg was significant versus control (3.5 ± 2.1 amebas/mm^2^, *P* < 0.05, *n* = 5). In addition, liver immunohistochemistry for 220 KDa in animals infected showed fragmented amebas in the granuloma area with fibrosis and inflammatory infiltrate. In contrast, animals with ALA treated with a low dose of EEO showed minor inflammatory infiltrate with trophozoites around hepatic sinusoid (Figure 5(b)). Whereas, animals treated with high dose of EEO had a scarce inflammatory infiltrate and less fibrosis (Figure 5(d)) compared to animals with ALA (Figure 5(b)). Therefore, the Ascaridole had an amebostatic effect that suggests a total reversion.

Some studies have reported that the administration of Epazote infusions or EEO can be lead to overdoses [11, 14]. However, the administration by oral route for seven days in this study showed to be safe, because there are no deaths among the animals. The result is in agreement with others that administrated EEO for oral route and long periods [17, 29]. On the other hand, Ascaridole partial effectiveness found in groups with ALA + EEO could be due to its pharmacokinetics because Ascaridole has been shown to have a half-life of 0.5 h, when administered orally (doses 30, 60, and 120 mg/kg) and it is removed rapidly from the blood stream [16].

The complete cure of the animals treated with the essential oil of Epazote did not occur totally. However, untreated animals develop the inexorable disease; the hamsters treated with the essential oil by oral route had small lesions and low parasite burden (Figures 3 and 5). The model of ALA due to* Entamoeba histolytica* is not a perfect model, because it is a highly virulent strain and causes a disseminating, ‘‘noncure,” and it carries on fatal diseases in hamsters. However, the efficacy partial to EEO produces a consistent protection against the* Entamoeba histolytica*.

Our results show that EEO has antiamoebic activity in vitro and in vivo. Since, the Ascaridole was detected by CG-MS as the major component of Epazote, we suppose that antiamoebic effect could be due to 1, 4 endoperoxide which has been found as the active component for anthelmintic and antimalarial effects [14, 27]. It is interesting to speculate about the mechanism by which Ascaridole kills trophozoites. It is an endoperoxide that it can deliver reactive oxygen species and damage the trophozoites in a similar way that oxygen peroxide induces toxicity to amoeba [30]. Recently, Monzote et al. [31] reported the toxic effects of* Chenopodium* essential oil and its components on some protozoa as* Leishmania*,* Trypanosoma brucei,* and* Trypanosoma cruzi*, and they concluded that the Ascaridole exhibited the better antileishmanial activity and suggest that the action mechanism through the generation of oxygen radicals, mitochondrial dysfunction, and a modification of redox indexes. This mechanism might not be applicable to amoebas, because they have not mitochondria; however, the free radical can be impact on their membrane electron transport system that serves to maintain in good condition the redox balance [32]. Alternatively, other targets of Ascaridole in the amoeba can be free radical-triggered DNA or protein alterations.

## 4. Conclusion

In this study, we have described the ostentation of EEO from Epazote. The Ascaridole is the main component of the essential oil. It exhibited antiamoebic activity in vitro and in vivo against* Entamoeba histolytica*. This report provides important information on biologically active terpenoid compounds found in traditional medicine plants as the Epazote.
